# The influence of maternal singing on well-being, postpartum depression and bonding – a randomised, controlled trial

**DOI:** 10.1186/s12884-021-03933-z

**Published:** 2021-07-12

**Authors:** Verena Wulff, Philip Hepp, Oliver T. Wolf, Tanja Fehm, Nora K. Schaal

**Affiliations:** 1 Department of Experimental Psychology, Heinrich-Heine-University Düsseldor, Universitätsstraße 1, 40225 Düsseldorf, Germany; 2 Clinic for Gynecology and Obstetrics, University Clinic, Augsburg, Germany; 3 University Witten/Herdecke, Landesfrauenklinik, Wuppertal, Germany; 4 Department of Cognitive Psychology, Institute of Cognitive Neuroscience, Faculty of Psychology, Ruhr-University Bochum, Bochum, Germany; 5 Clinic for Gynecology and Obstetrics, Heinrich-Heine-University Düsseldorf, Düsseldorf, Germany

**Keywords:** Maternal health, Mother-infant bonding, Music, Singing, Postpartum depression

## Abstract

**Background:**

Postpartum depression is fairly common in new mothers and moreover associated with impaired bonding and poor maternal well-being. The aim of the present study was to investigate the impact of a mother-infant singing intervention within the first three months after birth on maternal well-being, depressive symptoms and bonding.

**Methods:**

120 women who were recruited at the maternity ward at the University Clinic in Düsseldorf took part in this prospective, randomised-controlled study. Beside the baseline measurement 1–3 days after childbirth, depressive symptoms, maternal well-being and mother-infant bonding were evaluated with questionnaires before (two weeks after birth) and after (twelve weeks after birth) the intervention took place. The experimental group (*n* = 59) participated in several singing intervention sessions while the control group (*n* = 61) did not. In the intervention group, salivary cortisol as well as attachment and mood were assessed immediately before and after the singing sessions.

**Results:**

The participants of the intervention group showed a significant reduction of cortisol (*p* = .023) and an improvement of attachment and mood from start to end of the intervention session (all *p* ≤ .008). However, no prolonged effects were revealed beyond the intervention sessions as the two groups did not differ regarding the alterations of the primary outcomes postpartum depression (interaction effect *p* = .187) and postpartum bonding (interaction effect *p* = .188) in the 10-week period from two up to twelve weeks after childbirth (all *p* > .05). Additional analyses of singing habits at home in both groups, revealed that only in the singing group more frequent singing was associated with less anxiety and more well-being of the mother.

**Conclusion:**

Singing towards the infant seems to have positive immediate effects on the well-being of new mothers (on subjective variables as well as physiological measurements). However, the intervention did not lead to more long lasting positive effects although several limitations should be considered.

**Trial registration:**

DRKS00015178 (registered at the German Clinical Trial Registry), date of registration: 09.11.2018.

## Background

After birth, about half of new mothers suffer from temporary mood disturbances such as tearfulness, emotional lability, feelings of inability to cope with the baby as well as worries about the baby’s well-being, that are known as the "Baby Blues", which usually occur within the first week after birth and resolve after a few hours or several days [1, 2]. One reason for the mood disturbances after birth is the withdrawal of reproductive hormones that impact further systems in the brain like the hypothalamic-pituitary-adrenal axis and the limbic system that are associated with depression [3]. Normally, the biological systems regulate and stabilise over time but sometimes the disturbances remain and result in postnatal depression [3, 4]. In the case that the depressive symptoms last longer than two weeks, it is possible that a postpartum depression manifests [5]. Postpartum depression is not uncommon and about 10–15% of new mothers are affected by it within the first twelve months after childbirth. The highest prevalence for developing postnatal depression is up to the third month after childbirth and thereafter the prevalence decreases [4]. Women who have developed postpartum depression suffer from impaired mental and psychological health (like lower self-esteem, anxiety or emotional problems) as well as lower quality of life, less social relationships and an increased risk to develop addictive behaviors [6]. However, postpartum depression is not only a problem for the affected woman but can also have short- and long-term impact on the child. As a consequence of depression, the mother shows less interaction with the infant, less positive responsiveness to the infant’s affect and overall less positive emotions [7, 8]. This lack of response and interaction can have a substantial impact on the baby. Studies have shown that postpartum depression of the mother can cause eating and sleeping difficulties of the baby [7], influence the temperament of the infant [9] and the emotional tie between mother and baby [10] which is known as mother-infant bonding or attachment. Disturbances in parts of the attachment system (such as disturbed responsiveness, less interaction or flattened maternal emotions due to stress, anxiety or depression) can have a wide range of negative consequences such as impaired cognitive or emotional development that may last over years [11, 12]. Thus it is desirable to develop suitable interventions to improve postnatal maternal well-being in order to prevent negative consequences such as depressive symptoms and impaired mother-infant bonding.

In recent years, an increasing body of research showed beneficial effects of music interventions and music assisted relaxation techniques on arousal, stress and anxiety that are visible in psychological and physiological parameters [13–15]. Furthermore, beneficial effects of music were reported in clinical contexts, for example regarding the treatment of depression as Leubner and Hinterberger [16] demonstrated in their review of 28 studies. In sum, they found a positive impact of different kinds of music interventions like passive music listening, active singing and playing music or improvising on depression score improvements. Beyond that, positive effects of music have been reported in the context of childbirth [17]. A review of Lin et al. [18] summarises positive effects such as significant decreases of anxiety scores or improvements in physiological parameters like heart rate and blood pressure of the mother during labour. Furthermore, Nwebube, Glover and Stewart [19] showed that pregnant women who listened to special composed songs for pregnant women report lower anxiety and depression scores compared to a control group that conducted daily relaxation. In a study by Wulff et al. [20] the immediate and more prolonged effects of a music and a singing intervention were explored in pregnant women during the last trimester of pregnancy. Immediate improvements of salivary cortisol, oxytocin and maternal mood were found while the expectant mother listened to music or sang lullabies for the unborn baby. In addition to that, more prolonged effects of the interventions were reported for the perceived closeness to the baby and self-efficacy when compared to a control group.

For the time period after birth, only a few studies examined the effects of music on the emotional state of the mother. A review and meta-analysis of Yang et al. [21], summerised positive effects on postpartum depression and anxiety. Of the analysed seven studies that conducted daily music therapy sessions with a duration of a minimum of 30 min, the majority found positive effects on postpartum depressive symptoms and anxiety while one study reported additional positive effects on pain, sleep satisfaction and attachment. Although positive effects were found, the authors highlighted the heterogeneity of study designs and sample sizes and concluded that more research is needed to confirm the promising effects of music interventions after childbirth.

As a special form of making music, singing is used by mothers around the world as a tool to relax and calm the baby [22–24]. There is evidence that music and singing can affect bonding and interactions in social contexts, as well as possessing evolutionary aspects of infant care and parental attention [25–27]. It has been shown that directly after birth, infants have the ability to perceive and process complex musical stimuli [28, 29]. Additionally, maternal singing has the power to modulate the infant’s arousal and even its physiological state after birth [30–32]. The mother is able to adjust the state of the child for example through the singing style and can induce decreasing arousal as a response to soothing singing as well as to particular musical aspects of lullabies [33, 34]. In the course of this, studies showed that features of songs can indicate context and intention. For example lullabies can be identified as calming songs in the context of infant care independent of culture, familiarity or language [22, 34, 35]. They also seem to have an universal influence on infants´ states which indicates that there is a predisposed ability to respond to music [34].

In a non-obstretical setting, a positive impact of singing on affect and arousal was found in a sample of choir singers that was visible in a significant reduction of negative affect and a trend of salivary cortisol reduction in the singing compared to a non-singing group [36]. The release of cortisol is caused by an activation of the hypothalamus-pituitary-adrenal axis through the limbic system during stress and therefore it is often used as a physiological stress-marker that can be easily collected with saliva samples [37, 38] and shows decreases in the context of relaxing music and singing interventions [15, 36]. Overall, singing seems to promote health and well-being [39].

With regard to the time of pregnancy, Persico et al. [40] revealed positive effects of a prenatal singing intervention only on postnatal mother-infant bonding and neonatal crying episodes but no effects during pregnancy in comparison to a control group. Only a few studies explored the effect of maternal singing towards the baby in the postpartum period. Fancourt and Perkins [41] investigated the effect of mother-infant singing compared to playing with the baby on the emotional closeness to the infant and the affect of the mother. With a sample of 43 mother-infant-dyads with three - up to 14-month-olds, the study revealed in a within-subject design that singing in a 35 min musical workshop leads to a significant greater increase of emotional attachment to the baby and to a greater increase in positive maternal affect as well as to a larger decrease in cortisol when compared to a 35 min workshop without singing components. Another study, that was conducted with a sample of depressive mothers with their up to ten months old babies, compared a music group to a play group and a control group and investigated the impact on depressive symptoms with a ten-week-intervention program [42]. Women that participated in the music group sang together and learned new songs whereas the play group included creative and sensory play with the babies and the control group did not receive any kind of intervention. In case of higher depressive symptoms at baseline, women in the music group showed the fastest improvement after six weeks of workshop participation compared to the play and control group. No significant impact of group allocation was found for women with a medium score of depression at baseline.

Based on first promising findings that music and singing can have a positive effect on postnatal depressive symptoms of the mother [21, 42] as well as on mother-infant bonding [41], the aim of the present study was to investigate the effect of a music intervention (group singing with the infants) in comparison to a control group (no intervention) on some of the most relevant factors that are associated with maternal and infant health after birth in particular on maternal depressive symptoms, well-being and mother-infant bonding. In contrast to previous studies, the impact of singing was investigated in a randomised controlled trial design and for the first time, the effects on the subjective factors mother-infant bonding, depressive symptoms and maternal well-being as well as on salivary cortisol as a physiological marker for stress were explored within one study.

While a baseline measurement was conducted 48 h after childbirth (baseline) in order to control for group differences at the time of recruitment, a factorial design with two parallel arms was used to investigate the impact of a singing intervention in a randomised controlled study. Therefore, measurements took place before (two weeks postpartum (T1)) and after (12 weeks after childbirth (T2)) the intervention group participated in the intervention sessions, while the control group received no further care in this period. The effects on mother-infant bonding, depressive symptoms and maternal well-being were investigated with questionnaires. Additionally, in the intervention group saliva samples for cortisol determination were taken to investigate the immediate effect of the intervention session on a physiological stress parameter. In relation to previous findings regarding direct effects of music interventions [36, 41], an immediate positive effect was expected during the 45 min long sessions (from pre to post session) i.e. an increase of positive affect, a decrease of negative affect and a stress reduction visible through a cortisol reduction during the intervention. Furthermore, in line with previous studies [41, 42], it was expected that the singing intervention will have a positive effect on all variables and therefore lower despressive symptoms, higher mother-infant bonding as well as higher well-being at T2 were expected.

## Methods

### Sample

**Fig. 1:**
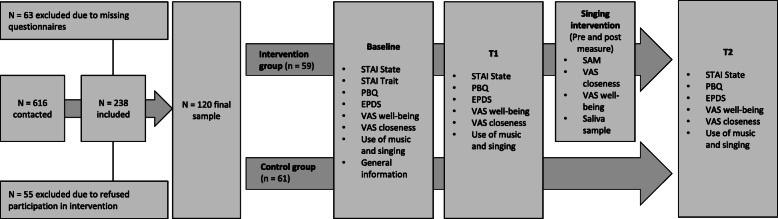
Flow chart of the sample. The final sample participated in all measurement time points

Between November 2018 and October 2019, study participation was offered to 616 new mothers within 48 h after childbirth at the Clinic for Gynecology and Obstetrics at the University Hospital Duesseldorf and 238 women agreed to take part in the study. A sample size calculation was conducted with the programm G*Power [43]. Based on an expected low to medium effect size (*f* = 0.15), a power of 80% and an alpha-error of 0.05 in the present study design, the required sample size was 176 participants (88 per group). We expected several drop-outs and therefore reached a sample of 238 participants. Due to a larger drop-out through missing questionnaires (*n* = 63) or refused attendance to the intervention sessions (*n* = 55), the final sample contains 120 women (see Fig. 1). A post hoc power analysis with G*Power with the given sample of 120 participants, a low effect size of *f* = 0.10 and a correlation between measures of *r* = .60 revealed a power of 68%. The participants were aged between 19 and 44 years (*M* = 33.73, *SD* = 4.74) and had a gestational age between 35 + 1 and 42 + 1 weeks (*M* = 38.81, *SD* = 1.62) at the time of childbirth. As criteria for inclusion, women had to be aged over 18 years, have sufficient knowledge of the German language and no serious comorbidities or pregnancy and birth complications. All pregnant women, who met our inclusion criteria, were offered participation. We did not screen patients regarding their level of depression or distress as the aim was to include the whole potential population and not only women with moderate or high levels of depression and distress. All participants received detailed information for study participation from a member of the study team on the maternity unit and gave informed written consent. Following that, they filled in the first questionnaire and the study team member allocated them to the control or intervention group via a computer-assisted permuted block randomisation (1:1 allocation ratio). Therefore, the study was not blinded as the participants as well as the team members were aware of the group allocation. The study was approved by the ethics committee of the Medical department of the Heinrich-Heine-University in Düsseldorf (Germany) and was registered in the “Deutsches Register Klinischer Studien” (DRKS00015178). The study adheres to CONSORT guidelines. No harms or unintended effects were revealed in this study.

### Questionnaires

To measure anxiety, the State-Trait-Anxiety Inventory [44] was used. The subscale *State* of the State-Trait-Anxiety Inventory was used to measure the temporary subjective feeling of anxiety and the subscore *Trait* was used to measure the general tendency toward anxiety. Each scale consists of 20 statements with response options on a 4-point likert scale from “almost never” to “almost always”. For analysis, an overall sum score was calculated for each subscale with a possible range from 20 to 80 for which a higher score indicates higher anxiety levels.

For the measurement of postpartum depression, the German version of the Edinburgh Postnatal Depression Scale [45] was used. The Scale consists of 10 statements and participants are asked to rate the appropriate feeling within the last 7 days on a 4-point likert scale from 0 to 3. A sum score was calculated which could range from 0 to 30. A higher score reflects more symptoms of depression and a higher probability to suffer from depression.

Mother-infant attachment was measured with the German version of the Postpartum Bonding Questionnaire [1] that consists of 16 items. Participants had to state the frequency of attachment-related emotions on a 5-point likert scale from “always” to “never”. The calculated overall sum score indicates the impairment of bonding with a higher score indicating greater impairment (possible range 0–80).

In addition to the Postpartum Bonding Questionnaire, mother-infant bonding was measured with a visual analogue scale where participants had to rate their perceived closeness to the baby with a cross on a 10 cm line. According to the actual subjective feeling, they were asked “How close do you feel to your baby?” (visual analogue scale closeness to the baby) and the response was given between the anchors “No closeness to the baby” on the left end and “Maximum closeness to the baby” on the right end. The score was measured in cm from the left end of the scale with a possible score between 0 and 10 cm. Another visual analogue scale was used to measure the perceived comfort with the maternal role (visual analogue scale comfort with maternal role). Participants had to rate their answer to the question “How comfortable do you feel with the maternal role?” between the anchors “Not comfortable” on the left end and “Maximum comfortable” on the right end.

Additionally, several questions about the use of music and singing were asked at each time of measurement. Participants were asked “How often did you sing for your baby since birth?” (frequency singing (baby)), “How often did you sing for yourself?” (frequency singing (oneself)), “How often did you play music for your baby since birth?” (frequency playing music (baby)) and “How often did you listen to music for yourself?” (frequency listening music (oneself)) and had to rate their answer on a 5-point likert-scale (“never”, “once per week”, “several times per week”, “once per day”, “several times per day”).

Furthermore, additional items were presented in the intervention group at T2 to inquire the satisfaction with the intervention. A visual analogue scale was used where participants had to rate their answer to the question “How satisfied were you with the intervention?” (visual analogue scale satisfaction with intervention) on a 10 cm line between “Not at all” and “Very satisfied”. They were also asked whether they would participate again with “Yes” and “No” as possible answers.

In order to assess the emotional state at the beginning and at the end of the first intervention session, participants filled in the Self-Assessment Manikin (SAM) [46]. The questionnaire has three items where participants rate their actual feeling regarding the dimension valence, arousal and dominance via visual figures. The figures depicture a range of each dimension on a 9-point likert-scale and participants are asked to mark the appropriate figure. There is a range of 5 figures with 4 possible interim points from “pleasant” to “unpleasant” for the valence dimension, from “excited” to “calm” regarding the dimension of arousal and from “dependent” to “independent” for the dominance dimension and each score has a range from 0 to 5. Higher scores indicate less pleasure, less arousal and a greater feeling of independence.

Maternal salivary cortisol was measured in order to evaluate a physiological marker for stress of the participants when they participated in the intervention session for the first time whereas no samples were taken from the infants. Cortisol is widely used as a biomarker in stress research due to its reflection of the activity of the sympathetic nervous system [37]. Saliva samples were taken with Salivettes (Sarstedt, Germany) at the beginning and at the end of the intervention session. Participants insalivated cotton swabs for at least 30 s. After the samples were taken, they were stored at − 18 °C until further analysis. Cortisol levels were determined in the laboratory of the DresdenLAB (Dresden, Germany) by using immunoassay (IBL, Hamburg, Germany).

### Procedure

Eligible women were visited on the maternity unit within 48 h after childbirth and participation was offered to them. An information sheet was given to the participants containing information that prior studies showed positive effects of singing on several aspects of well-being and therefore the intervention was designed to explore effects of singing in the postpartum context. In this course, it was not explicitly mentioned on which outcomes effects were expected. After informed written consent was obtained, they were randomised into the control or intervention group and received the baseline questionnaire that comprised the State-Trait-Anxiety Inventory *State* and *Trait*, Postpartum Bonding Questionnaire, Edinburgh Postnatal Depression Scale, visual analogue scales about well-being and closeness to the baby as well as questions about the use of music and singing in paper-pencil format. Some general information such as age and gestational age was taken from the medical record. A member of the study team returned a few hours later to collect the completed questionnaire and to give a pair of baby socks away as a thank you for participation. Two weeks after the baseline measurement, participants received an invitation with a link to fill in the first questionnaire (T1) that contained the State-Trait-Anxiety Inventory Stubscore *State*, Postpartum Bonding Questionnaire, Edinburgh Postnatal Depression Scale, visual analogue scales about well-being and closeness to the baby as well as questions about the use of music and singing via the online-platform SoSci-Survey [47] with the request to complete the survey within the next three days. In case of a missing completion, up to two reminders were sent. Participants of the intervention group made appointments for the intervention sessions during the following weeks. They were asked to take part at least once between the third and the 10th week of the baby’s life so that the starting point of the intervention period varied slightly between participants. Women of the intervention group had the possibility to take part in the sessions with their babies up to three times with the latest possibility of participation in the 12th week after birth. The control group only received standard care, which does not include any mental health care or screening from hospital staff. At T2 (12 weeks postpartum), all participants received an e-mail with the link to the second online questionnaire that contained the same questionnaires as T1 and with a request for completion within three days. Reminders were also sent in case of missing completion. As soon as a woman completed T2, a baby rattle was sent to them as a thank you for study participation. In the case that participants reached cut-off scores for clinical relevance in the questionnaires, they were informed by phone and information about contact points and possibilities for check ups (gynaecologist, hospital staff, and midwives) were given. An overview of the procedure as well as information about when which questionnaire was administred is given in Fig. 1.

The intervention session took place every second week in a gymnastic room of the Clinic for Gynecology and Obstetrics at the University Hospital Duesseldorf and the women that were randomised into the intervention group participated between one and three times. Five to ten women participated simultaneously in one intervention session. When the participants arrived with their babies, they were welcomed by a member of the study team and one music therapist that moderated the intervention class. All participants filled in the pre intervention session questionnaire that comprised the Self-Assessment Manikin and the visual analogue scales about well-being and closeness to the baby insalivated a saliva sample. After a short welcome that always contained a “welcome song” where all participants and infants were addressed with names, the concept of the intervention was explained. Due to the explanations given, the participants were aware of the goal of the intervention, which was to implement a singing- and music-based interaction between the new mother and the baby at home. Therefore, elements of finger games, lullabies and movements to music were used and all participants practiced them together with their infants during the intervention session which lasted 45 min. Although a standard repertoire of songs and games existed, the music therapist incorporated the participants´ requests and wishes if present. The participants were asked to implement the intervention daily at home. At the end of the intervention session, all participants filled in the questionnaire again and insalivated a second saliva sample.

### Statistical analysis

For the statistical analysis, the statistical software package SPSS 24 (IBM Inc., Armonk, NY) was used. When sphericity was violated, Greenhouse-Geisser corrected values were reported. Only the data of participants that performed by protocol were used for calculations (i.e. participants who were randomized into the intervention group but did not attend the intervention, were excluded from the analysis). Outliers above two standard deviations from the mean, were excluded separately for each calculation. The maximal number of excluded outliers was six per measure. This outlier correction did not affect the results in a substantive way. In case of up to two missing values, replacements with the mean scores of the norm sample were conducted for the State-Trait-Anxiety Inventory as suggested by Laux et al. [44] and for the other questionnaires with the sample mean for each item [48]. Furthermore, group differences at baseline were checked with t-tests for independent samples regarding maternal age, gestational age and State-Trait-Anxiety Inventory *Trait*.

In order to explore the immediate effects during the intervention session, dependent-sample t-tests were used to compare pre-post differences for the dependent variables Self-Assessment Manikin valence, arousal and dominance as well as for saliva cortisol. For the anaylsis of the immediate effects of the intervention on the three dimensions of the Self-Assessment Manakin (valence, arousal and dominance) as well as on salivary cortisol, only the data of the first intervention session for each women were used. All women in the intervention group had immediate effects data.

To investigate 10-week effects over the two times of measurement (T1 and T2), 2 × 2 mixed factorial ANOVAs with the independent variable *group* (control vs. intervention group) and the repeated-measure variable *timepoint* (T1 and T2) were applied with the dependent variables Trait-Anxiety Inventory Stubscore *State*, Edinburgh Postnatal Depression Scale, visual analogue scale comfort with the maternal role, visual analogue scale perceived closeness to the baby and Postpartum Bonding Questionnaire respectively.

In order to check for normality, Shapiro-Wilk tests were calculated for all dependent variables. The Shapiro-Wilk tests revealed that most variables were not normally distributed (*p* > .05). Even though the normality assumption was violated, all analyses were conducted as intended because of the proved robustness of ANOVAs [49, 50] and the absence of appropriate non-parametric alternatives for repeated-measures ANOVAs.

As explorative analysis, Mann-Whitney U tests were conducted in order to check for group differences between the intervention and the control group regarding the ordinal scaled frequencies of the use of music and singing at T2. Additionally, the relations between the frequencies of the use of music and singing and the variables at T2 (State-Trait-Anxiety Inventory *State*, Edinburgh Postnatal Depression Scale, visual analogue scale comfort with the maternal role, visual analogue scale closeness to the baby and Postpartum Bonding Questionnaire) were explored with Spearman correlations. Furthermore, Fisher’s z tests were conducted to examine group differences for the correlations. No corrections for multiple comparisons were applied due to the explorative character of the calculations [51].

## Results

### Sample characteristics

From the final sample (*N* = 120), 59 women were in the experimental group and 61 were in the control group. No group differences were revealed regarding maternal age, gestational age at the time of childbirth and State-Trait-Anxiety Inventory *Trait* (*p-values* ≥ .394) at the start of measurements (baseline). In regard to the relevant dependent variables (State-Trait-Anxiety Inventory *State*, Edinburgh Postnatal Depression Scale, visual analogue scale comfort with the maternal role, visual analogue scale closeness to the baby and Postpartum Bonding Questionnaire) no group differences were found at baseline (*p-*values ≥ .277). See Table 1 for the descriptive statistics and the p-values of the test-statistics regarding group differences at baseline.

**Table 1:** Sample characteristics. Descriptive statistics (means (standard deviations)) and results of calculations of group differences (p-values) at baseline

	Control group			Intervention group			
	M	SD	n	M	SD	n	p-value
Age^A^	34.31	(4.19)	61	33.67	(3.94)	58	*p* = .394^C^
Gestational Age^B^	38.76	(3.67)	61	39.05	(1.51)	57	*p* = .713^C^
STAI Trait	47.00	(5.25)	60	46.53	(4.85)	53	*p* = .627^C^
STAI State	36.06	(9.89)	58	36.63	(9.10)	54	*p =* .753 ^C^
EPDS	7.13	(4.34)	56	6.36	(4.04)	54	*p* = .341 ^C^
VAS comfort with the maternal role	8.59	(1.25)	57	8.58	(1.15)	53	*p* = .970 ^C^
VAS closeness to the baby	9.49	(0.65)	55	9.34	(0.82)	53	*p* = .277 ^C^
PBQ	5.51	(4.12)	54	5.14	(3.98)	54	*p* = .630 ^C^

Note. ^A^ = in years at the time of childbirth, ^B^ = in weeks at the time of childbirth, ^C^ = result of an independent-samples t-test. STAI: State-Trait-Anxiety Inventory; EPDS: Edinburgh Postnatal Depression Scale; VAS: visual analogue scale; PDQ: Postpartum Bonding Questionnaire; n = participants included in the analysis

### Immediate effects during the intervention

**Fig. 2:**
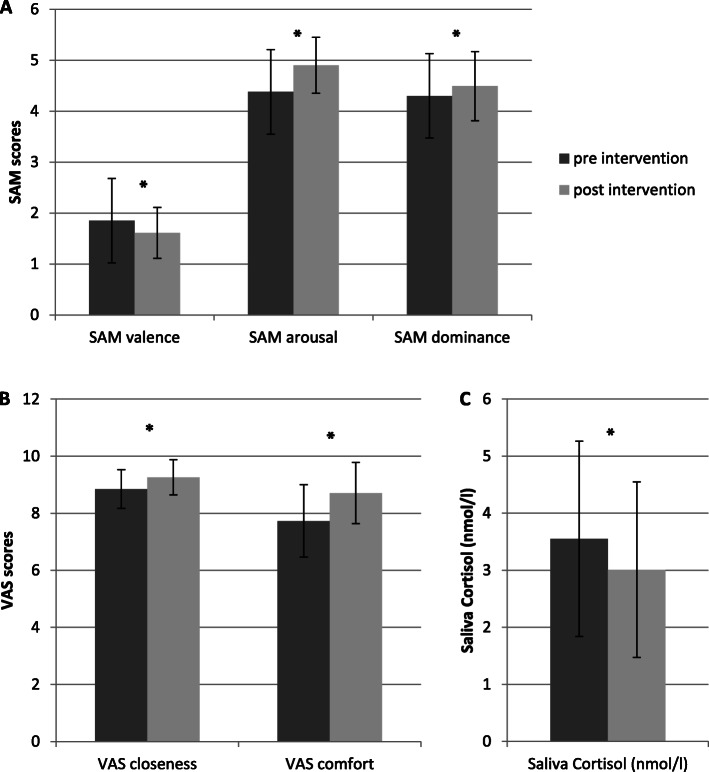
Results of the immediate effects during the 45-min intervention session with a comparison between pre and post intervention measurement; * = *p* < .05; error bars represent standard deviations. **A**: Results of the SAM scores valence, arousal and dominance. A significant improvement was found from pre to post intervention for all dimensions of SAM. **B**: Results of the VAS scores closeness to baby and comfort with maternal role. Both scores increased significantly from pre to post intervention, indicating an improvement of bonding and comfort. **C**: Comparison of salivary cortisol levels between pre and post intervention session. Saliva cortisol decreases significantly during the intervention session

In the context of the intervention session, dependent-sample t-tests showed significant improvements of the emotional state from pre to post measurement (see Table 2 for the descriptive statistics and the number of participants included in each analysis). Regarding the Self-Assessment Manikin valence score, a significant difference from pre to post was revealed [*t*(50) = 3.33, *p* = .002, d = 0.43 (CI 95% 0.03, 0.82)] with lower scores at the end of the intervention indicating more happiness at the end of the session compared to the start. A significant difference was also revealed for the Self-Assessment Manikin arousal score [*t*(49) = − 5.11, *p* < .001, d = 0.65 (CI 95% 0.25, 1.05)] that increased during the intervention session which indicates less arousal at the end of the intervention session. The difference between the pre and post measurement regarding Self-Assessment Manikin dominance score was also significant [*t* (52) = − 2.76, *p* = .008, d = 0.37 (CI 95% -0.01, 0.76)]. The participating women reported a higher feeling of independence and self-confidence at the end of the intervention session (see Fig. 2a).

**Table 2:** Descriptive statistics (mean values and in parentheses standard deviations) of the variables that were measured during the music intervention session (pre and post)

	Pre intervention	Post intervention	p – value^A^	n
SAM valence	1.85 (0.58)	1.61 (0.50)	.002	51
SAM arousal	4.38 (0.77)	4.90 (0.55)	< .001	50
SAM dominance	4.30 (0.75)	4.49 (0.68)	.008	53
VAS perceived closeness to the baby	8.85 (1.08)	9.26 (0.73)	.001	52
VAS comfort with maternal role	7.73 (1.27)	8.71 (1.07)	< .001	49
Saliva Cortisol (nmol/l)	3.55 (1.71)	3.01 (1.54)	.023	50

Note. ^A^ = t-tests for dependent samples for the comparison between pre and post intervention. SAM: Self-Assessment Manikin; VAS: visual analogue scale; n = participants included in each analysis

Furthermore a significant difference between pre and post measurement was revealed for the score of visual analogue scale *perceived closeness to the baby* [*t* (51) = − 3.40, *p* = .001, d = 0.43 (CI 95% 0.04, 0.82)]. After the intervention session, women reported higher feelings of closeness and attachment. The score of visual analogue scale *comfort with the role of the mother* also differed from pre to post measurement [*t* (48) = − 5.59, *p* < .001, d = 0.74 (CI 95% 0.33, 1.15)]. At the end of the intervention session, women reported more feelings of well-being and confidence with the maternal role (see Fig. 2b).

For salivary cortisol, a t-test for dependent samples showed a significant difference between the pre and post measurement [*t* (49) = 2.35, *p* = .023, d = 0.32 (CI 95% 0.07, 0.72)]. The cortisol level decreased significantly during the intervention session (see Table 3).

**Table 3:** Descriptive statistics (means (standard deviations)) of the variables that were measured at T1 and T2. Comparison between the control group and the intervention group

	T1						T2					
	CG		n	IG		n	CG		n	IG		n
STAI State	37.30	(7.01)	57	36.04	(8.04)	56	33.44	(5.92)	57	33.36	(6.71)	56
EPDS	5.05	(4.09)	56	5.37	(3.55)	54	4.25	(3.38)	56	3.72	(2.95)	54
VAS comfort with the maternal role	8.01	(1.38)	56	7.98	(1.31)	56	8.17	(1.35)	56	8.32	(1.12)	56
VAS closeness to the baby	9.30	(1.02)	56	9.55	(0.61)	52	9.55	(0.67)	56	9.50	(0.66)	52
PBQ	6.82	(5.02)	57	6.63	(5.16)	56	6.04	(4.40)	57	6.77	(4.48)	56

Note. CG = control group, IG = intervention group. STAI: State-Trait-Anxiety Inventory; EPDS: Edinburgh Postnatal Depression Scale; VAS: visual analogue scale; PDQ: Postpartum Bonding Questionnaire. N = participants included in each analysis

### 10-week effects

A mixed-factorial ANOVA with the dependent variable State-Trait-Anxiety Inventory *State* showed a significant main effect of the factor *time of measurement* [*F* (1,111) = 30.61, *p* < .001, d = 0.49 (CI 95% 0.22, 0.75)] with both groups showing a reduction of State-Trait-Anxiety Inventory *State* scores from T1 to T2. The main effect of the factor g*roup* was not significant [*F* (1, 111) = 0.33, *p* = .566, d = 0.11 (CI 95% -0.26, 0.48)] as well as the interaction effect [*F* (1, 111) = 0.99, *p* = .32, d = 0.19 (CI 95% -0.19, 0.56)]. See Table 2 for the descriptive statistics.

With a mixed-factorial ANOVA with the dependent variable Edinburgh Postnatal Depression Scale, a significant main effect for the factor *time of measurement* was revealed [*F* (1, 108) = 14.81, *p* < .001, d = 0.34 (CI 95% 0.07, 0.60)] and a reduction of depressive symptoms was visible from T1 to T2. No significant main effect of the factor *group* [*F* (1, 108) = 0.03, *p* = .859, d = 0.03 (CI 95% -0.34, 0.41)] and no significant interaction effect [*F* (1, 108) = 1.76, *p* = .188, d = 0.25 (CI 95% -0.12, 0.63)] were revealed.

A mixed-factorial ANOVA with the dependent variable visual analogue scale *comfort with the maternal role* showed a significant main effect of the factor *time of measurement* [*F* (1, 110) = 4.82, *p* = .030, d = 0.19 (CI 95% -0.07, 0.46)] but no significant main effect or the factor *group* [*F* (1, 110) = 0.06, *p* = .805, d = 0.05 (CI 95% -0.32, 0.42)] or an interaction effect [*F* (1, 110) = 0.65, *p* = .422, d = 0.16 (CI 95% -0.21, 0.53)] were revealed. Both groups showed an increase of comfort from T1 to T2.

The analysis of visual analogue scale *closeness to the baby* showed no significant main effect of the factor *time of measurement* [*F* (1, 106) = 1.91, *p* = .170, d = 0.13 (CI 95% -0.13, 0.40)]. The main effect of the factor *group* was also non significant [*F* (1, 106) = 0.60, *p* = .442, d = 0.15 (CI 95% -0.23, 0.53)] but a significant interaction effect was revealed [*F* (1, 106) = 4.19, *p* = .043, d = 0.97 (CI 95% 0.57, 1.37)]. However, post-hoc analyses showed no significant group difference at T1 [t (91.18) = 1.54, *p* = .127, d = 0.30 (CI 95% -0.08, 0.68)] or at T2 [t (107) = − 0.39, *p* = .695, d = 0.06 (CI 95% -0.32, 0.44)].

For the Postpartum Bonding Questionnaire no significant main effect of the factor *time of measurement* was revealed [F (1, 111) = 0.85, *p* = .359, d = 0.08 (CI 95% -0.18, 0.35)]. Furthermore, no significant main effect for the factor group [F (1, 111) = 0.104, *p* = .748, d = 0.06 (CI 95% -0.31, 0.43)] nor a significant interaction effect [F (1, 111) = 1.76, *p* = .187, d = 0.25 (CI 95% -0.12, 0.62)] was revealed.

### Further analysis

In order to investigate whether the group allocation is related to the frequencies of singing and listening to music at the second time of measurement (T2), Mann-Whitney U tests were performed. For the frequency *singing (baby)* no significant relation was found [*U* = 1625.50, *z* = −.99, *p* = .324]. However the group allocation was significantly related to the frequency of *singing (oneself)* [*U* = 1434.00, *z* = − 2.00, *p* = .045], the frequency of *playing music (baby)* [*U* = 1232.50, *z* = − 3.07, *p* = .002] and to the frequency of *playing music (oneself)* [*U* = 1390.50, *z* = − 2.20, *p* = .028]. Regarding these dependent variables, the intervention group reported higher frequencies of singing and music compared to the control group but overall, the majority of mothers reported the use of singing and music in everyday life independent of group allocation. See Table 4 for an overview of the descriptive statistics (mean rank scores).

**Table 4:** Descriptive statistics (absolute values of the frequencies of singing and playing music for onself and for the baby) at the second time of measurement (T2), separately listed for the intervention and the control group. Mean rank scores are additionally listed for interpretion of the Mann-Whitney U tests

		Frequency singing (baby)	Frequency singing (oneself)	Frequency playing music (baby)	Frequency listening music (oneself)
IG	never	1	17	1	8
	once per week	1	15	5	11
	several times per week	13	15	17	19
	once per day	13	6	20	12
	several times per day	31	6	16	9
	*M*_*rank*_	63.45	66.69	70.11	67.43
CG	never	1	29	9	19
	once per week	3	12	12	15
	several times per week	17	13	16	11
	once per day	12	2	15	7
	several times per day	28	5	9	9
	*M*_*rank*_	57.65	54.51	51.20	53.80

Note. IG = intervention group (n = 59), CG = control group (n = 61)

In order to explore the relation between the frequencies of listening to music or singing and the dependent variables at T2, correlations were calculated. Due to the group differences regarding the mentioned frequencies, Spearman correlations were conducted separately for each group. The results of the correlations are displayed in Table 5 as well as the between group comparison with Fisher’s z tests. It should be highlighted that in the intervention group, a higher frequency of singing for the baby was significantly associated with less maternal anxiety (State-Trait-Anxiety Inventory *State*) and with greater comfort with the maternal role (visual analogue scale). Additionally in the intervention group, the frequency of singing for oneself is significantly correlated with the Edinburgh Postnatal Depression Scale, visual analogue scale comfort with maternal role and Postpartum Bonding Questionnaire. The correlations indicate that a higher frequency of singing for oneself is associated with less depressive symptoms, greater comfort and closer bonding.

**Table 5:** Results of the correlations (Spearman correlation coefficients r) between the frequencies of listening to music and singing with the dependent subjective variables at T2. Results of the comparison between the intervention group and the control group are listed with Fisher’s z

		n	Frequency singing (baby)		Frequency singing (oneself)		Frequency playing music (baby)		Frequency listening music (oneself)	
STAI State	IG	58	- .26	*	- .24		- .23		- .10	
	CG	58	.05		- .39	**	- .16		- .20	
	*z*		−1.65	*	0.88		−0.38		0.53	
EPDS	IG	56	- .08		- .28	*	- .08		- .22	
	CG	57	.12		- .23		- .14		- .15	
	*z*		0.99		−0.28		0.33		−0.33	
VAS comfort maternal role	IG	57	.35	**	.41	**	.37	**	.24	
	CG	58	.02		.31	*	.11		.05	
	*z*		1.81	*	0.62		1.42		0.99	
VAS closeness to the baby	IG	54	.14		.21		- .07		.14	
	CG	60	.20		.13		.15		.04	
	*z*		−0.34		0.42		−1.12		0.52	
PBQ	IG	57	- .19		- .29	*	- .23		- .33	*
	CG	59	.01		- .08		- .06		- .07	
	*z*		−1.04		−1.16		−0.91		−1.40	

Note. IG = intervention group, CG = control group, * *p* < .05, ** *p* < .01. STAI: State-Trait-Anxiety Inventory; EPDS: Edinburgh Postnatal Depression Scale; VAS: visual analogue scale; PDQ: Postpartum Bonding Questionnaire

Additional analyses of the variables regarding the satisfaction with the intervention revealed that the majority of mothers would participate in the intervention lessons again (88.33%). Moreover the participants of the intervention group were very pleased with the intervention showing high scores of visual analogue scale pleasure with intervention (M = 8.47, SD = 1.98). The majority of the intervention group participated twice in the lessons (62.71%) whereas some women even participated three times (10.17%) and 27.12% of the intervention group participated only once.

## Discussion

In the present study, the effects of a singing intervention for mothers with their infants in the first weeks after birth were explored. Immediate effects of the singing intervention were found from pre to post intervention session. The data revealed a significant immediate reduction of cortisol levels as well as an immediate improvement of maternal scores on valance, arousal and dominance and mother-infant attachment (see Fig. 2), highlighting a significant positive immediate effect of the intervention on maternal stress levels, emotional state and bonding. However, no impact of the intervention could be revealed for the 10-week period between the second and the 12th week after birth. Regarding this time window, only time-effects but no group or interaction effects were revealed for the variables measuring maternal well-being.

Significant immediate positive effects were found from pre to post intervention session on affect, well-being and attachment in accordance with the hypotheses and previous studies [36, 41]. Regarding the Self-Assessment Manikin scores, significant improvements were found for valence, arousal and dominance showing that women were happier, more relaxed and more self-confident at the end of the intervention session compared to the beginning. The stress-reducing effect was also evident in the physiological stress marker salivary cortisol that decreased significantly during the intervention session. The improvement of well-being, mood and relaxation through music and singing is in line with other studies that showed similar improvements in other contexts [15, 36, 39]. The results are also in accordance with results of Fancourt and Perkins [41], who showed beneficial positive effects of a singing workshop for mothers and babies on maternal positive affect, cortisol and additionally on perceived closeness to the baby. Likewise, positive effects on attachment (visual analogue scale perceived closeness) and maternal well-being (visual analogue scale comfort with maternal role) were found in the present study with significant improvements. In accordance with our hypotheses, the maternal well-being and the attachment towards the baby seems to be positively affected by the music assisted interaction during the intervention session. Like Fancourt and Perkins [41] showed in their study, a music and singing intervention seems to encourage mother-infant bonding. Besides the mood improving and relaxing effects that were reported for singing in general [36, 39], singing also impacts social mechanisms. While feelings of social connectedness, social flow and bonding can be enhanced in particular by group singing [52, 53], similar social mechanisms seem to work during the interventions of the present study through the interaction with the newborn. In the initial period after birth, the variety of interaction is very restricted and during the first weeks the interaction is dominated by caring for the substantial infant’s needs and in particular (breast-) feeding [54]. Beyond that, singing can be an additional way to interact with the baby and leads to a stronger bonding between mother and child [20, 31]. Furthermore it is possible to induce infant feedback through singing like attention towards the mother or relaxation [31, 55]. This hypothesis is reinforced by the individual feedback of the participants who reported for example that they were “more sensitive in regard to interaction”, “interacted more with the baby” and “experienced a way to entertain the child” due to the intervention. One limitation is that the variables that were captured during the intervention (Self-Assessment Manikin, visual analogue scale closeness to the baby, visual analogue scale comfort with the maternal role, salivary cortisol) were not measured in the control group. Thus the immediate effects from pre to post intervention should be carefully interpreted because a comparison with the control group is not possible. In this respect, it would also be desirable to include an active control group (i.e. a playgroup without music elements) in future studies, as it may be that the positive immediate effects of the music group were influenced by the social experience of a group intervention.

In contrast to our hypotheses that the singing intervention would also show positive 10-week effects from T1 to T2, both groups showed similar reductions in State-Trait-Anxiety Inventory *State* scores and Edinburgh Postnatal Depression Scale scores. The alternation over time is in line with the fact that in the postpartum period, symptoms of anxiety and depression are common but decrease over time [56, 57]. Overall, the sample of the present study showed Edinburgh Postnatal Depression Scale scores (see Table 2) that are much lower than the cut-off score (EPDS ≥13) for a high probability of depression [45] while the State-Trait-Anxiety Inventory *State* comply with the scores of the German norm sample [44]. This indicates that the women of the present sample were in a good mental state and therefore little room for improvement was evident. As Fancourt and Perkins [42] revealed, the positive effects of the singing intervention were only visible in a subgroup of women with Edinburgh Postnatal Depression Scale scores ≥13, it may be hypothesised that women in a good mental health might not further improve their mood with an additional intervention. Beyond that, the additional exploratory analysis regarding the frequencies of the use of singing and music revealed no relation between group allocation and the frequency of singing for the baby showing that both groups sang for their babies in a similar amount which could explain the missing 10-week impact of the singing intervention during the time window from T1 to T2. Furthermore, a significant time effect but no group or interaction effects occured for the visual analogue scale *comfort with the maternal role* where high scores at all times of measurement (see Table 2) also indicate a ceiling effect. No influence of the intervention was found, which is also in contrast to our main hypotheses.

With regard to mother-infant bonding, a significant time effect was only revealed for the Postpartum Bonding Questionnaire, but, against our hypotheses, no group or interaction effect was found showing that the intervention did not influence mother-infant bonding measured with the Postpartum Bonding Questionnaire in the time frame from T1 to T2. The Postpartum Bonding Questionnaire scores decreased over time indicating an improvement of bonding, whereas the visual analogue scale *perceived closeness* scores remained stable in a high range. Although the interaction effect turned out significant for the visual analogue scale, the post-hoc t-tests showed no significant group effect at both time points. The significant interaction can be traced back to a slight descriptive group difference at T1, although it was not significant. The result of an increase in mother-infant bonding over time, which was visible in the Postpartum Bonding Questionnaire scores, is in line with results of O’Higgins, Roberts, Glover and Taylor [58] who observed stronger bonding along with time after childbirth. The lack of an observable impact of the intervention on attachment can also be explained with a ceiling-effect because all participating women showed strong mother-infant attachment at both times of measurements (see Table 2). In particular the Postpartum Bonding Questionnaire scores are even lower than the scores reported by Reck and colleagues [1] for non-depressed (*M* = 7.33, *SD* = 6.14) postpartum women. It has to be noted that in the current study participation was offered to the whole potential population of pregnant women without a pre-screening for depression or risk for bonding impairment in order to include a sample which is representative of the general (pregnant) population. We would hypothesise that related to the results regarding a greater improvement of depression in clinical samples [42], the impact of the intervention on bonding could be also greater in a high-risk sample with mothers who have an impaired attachment towards the infant. This hypothesis should be explored in future studies in which the effects of singing interventions should be investigated particular in mothers who report a very low and impaired mother-infant attachment.

Besides the potential ceiling effects, it is possible that the intervention period was too short because the women participated in the intervention only once to a maximum of three times. In contrast to the study of Fancourt and Perkins [42], where the mothers participated over 10 weeks in the intervention lessons, the intervention of the current study was less frequent. Overall, 55 women were excluded from the analysis because they refused to participate in the intervention. Unfortunately, we did not evaluate why participants refused to take part in the intervention sessions. However, it would be interesting to try to evaluate this in future studies, as it would provide valuable information for planning and improving study protocols in the future, in order to make participation in the early postpartal phase more convenient. In this respect, another limitation of the study is that the time point where the women took part for the first time varied between the second and the ninth week of the baby’s life. As a result, a later participation reduced the intervention period. Although, we made sure that the number of attended sessions were comparable (one to three attended sessions), the intervention period until the post-intervention measurement varied. Future studies should try to optimize the standardisation of the time window. Furthermore, we hypothesise that the intervention period may also have been too early. In the first weeks of life the infant’s feedback and interaction repertoire is very restricted which can lead to maternal dissatisfaction during interaction processes due to a reciprocal dependency of reactions [59, 60]. A missing or limited feedback of the baby during the intervention or at home could have led to a perception of unsatisfied interactions. Larger effects might be visible when the intervention takes place later as done in other studies [41, 42] where the infants´ age was a few months up to almost one year. In spite of these limitations and the missing significant effects, the majority of participants reported that they would like to participate again (88.33%) and the mean visual analogue scale *satisfaction with intervention* score was very high (*M* = 8.47, *SD =* 1.98). Another limitation is that the post-hoc power analysis revealed a power of 68% in the current sample of 120 participants, which is lower than the power of 80% that was presumed in advance. This should be kept in mind when interpreting the results. Additionally, it would be desirable if future studies would replicate the present findings with an increased sample size and power.

The exploratory analyses that were performed beyond the main hypotheses revealed that the intervention group used music and singing for oneself more frequently in every day life even though the group differences regarding singing for the baby were not significant. Although the main goal of increasing singing to the baby can therefore not be supported, the women who participated in the intervention sang significantly more frequently for themselves and additionally, used music more often as well as they played music for their babies more regularly in comparison to the control group. This highlights that the overall musical activity was increased by the intervention. The difference regarding the frequency of singing for the baby was not significantly related to the group allocation which can be explained by the common use of singing lullabies to calm a baby that is widespread almost all over the world [22]. This could also explain the absent impact of the intervention in the 10-week period. Unfortunately, it is not possible to restrict singing or playing music in the control group which in turn restrains the interpretation of the results of the comparison between groups and should be taken into account as a limitation. It is possible that the intervention did not specifically influence the frequency of singing for the baby, but led to an increased sensitivity to include music and singing in everyday life as well as an enriched musical environment at home. It would be interesting to investigate musical activities in everyday life in depth as part of further studies.

A correlation analysis revealed that the frequency of singing and playing music is significantly associated with relevant variables measuring well-being and attachment at the second time of measurement. The significant group difference regarding the relation between singing for the baby and anxiety (State-Trait-Anxiety Inventory *State*) and maternal well-being (visual analogue scale comfort with maternal role) highlighted that only in the intervention group more frequent singing for the baby was associated with less anxiety and greater well-being. We assume that the intervention group was more sensitive and aware to the use of singing and music as they received instructions to do so in the intervention sessions. The music therapist gave several examples for the practical use at home and underlined the aspects of relaxation, consciousness and pleasure that are relevant for the effects of music [61, 62]. This could have made the use of music and singing “more effective” and led to greater associations in the intervention group even though no 10-week effects of the intervention were revealed.

## Conclusions

In sum, the present study showed that an early postpartum singing-based intervention led to positive immediate effects as significantly lower cortisol levels as well as an improved maternal emotional state and mother-infant attachment could be revealed post compared to pre intervention sessions. Regarding 10-week effects from the second to the 12th week after birth, the study did not reveal any significant differences in maternal wellbeing, depressive symptoms and mother-infant attachment between groups. However, correlation analysis revealed that the frequencies of the use of music and singing at home were associated with improved wellbeing and mother-infant attachment independent of group allocation. Overall, the promising immediate effects and the correlation analysis highlight that early postnatal singing sessions could be a simple, cost-effective and well accepted possibility to improve maternal well-being and attachment in the early stages of motherhood which should encourage future research.

## Data Availability

The dataset is openly available at https://osf.io/hfx6w/
